# Attention-deficit/hyperactivity disorder and snuff use prior to and early in pregnancy

**DOI:** 10.1007/s00737-026-01742-x

**Published:** 2026-07-01

**Authors:** Anneli Andersson, Sofi Oskarsson, Ralf-Kuja Halkola, Zheng Chang, Brian D’Onofrio, Henrik Larsson, Catherine Tuvblad

**Affiliations:** 1 School of Behavioural, Social and Legal Sciences, Örebro University, Örebro, Sweden; 2 Karolinska Institute, Stockholm, Sweden; 3 Indiana University Bloomington, Bloomington, USA; 4 School of Medical Sciences, Örebro Univeristy, Örebro, Sweden

**Keywords:** ADHD, Snuff use, Pregnancy

## Abstract

**Background:**

ADHD is a neurodevelopmental disorder linked to impulsivity and self-regulation difficulties. While the association between ADHD and nicotine dependence is well-established, less is known about the association with smokeless tobacco use, such as snuff. Given the adverse health effects of nicotine during pregnancy, this study examines whether women diagnosed with ADHD are more likely to use snuff prior to and early in pregnancy, and whether they persist in use between these periods. This study also explores the associations between ADHD and snuff use in the presence of common psychiatric disorders: depression, anxiety and substance use disorders (SUDs).

**Methods:**

Using Swedish population-based registers, we identified women who gave birth between 2000 and 2020. ADHD was defined based on clinical diagnoses and/or ADHD medication prescriptions. Snuff use was self-reported during a prenatal care visit. Logistic regression models estimated odds ratios (ORs) with 95% confidence intervals (CIs) for snuff use prior to and early in pregnancy among women with ADHD, adjusting for sociodemographic factors. We also stratified the analyses by depression, anxiety, and/or SUDs to assess potential differences in associations.

**Results:**

Women diagnosed with ADHD were more likely to use snuff prior to pregnancy (adjOR = 1.89, 95% CI: 1.83–1.95), early in pregnancy (adjOR = 2.43, 95% CI: 2.32–2.55), as well as be persistent snuff users (adjOR = 2.39, 95% CI: 2.27–2.52) compared to women without ADHD. Stratification by common psychiatric disorders revealed that the associations between ADHD and snuff use prior to and early in pregnancy were strongest among women diagnosed with ADHD without depression, anxiety, and/or SUDs.

**Conclusions:**

ADHD is an important risk factor for snuff use prior to and early in pregnancy, underscoring the need for targeted interventions to prevent nicotine use in young women with ADHD as part of integrated care.

**Trial registration:**

Retrospectively registered.

**Supplementary Information:**

The online version contains supplementary material available at https://doi.org/10.1007/s00737-026-01742-x.

## Introduction

Attention-Deficit/Hyperactivity Disorder (ADHD) is a common neurodevelopmental disorder with a strong genetic basis (Faraone and Larsson 2019). It typically manifests in childhood and often persists into adulthood, affecting various aspects of daily functioning (Polanczyk et al. 2007; Kessler et al. 2006). Individuals with ADHD are at least 1.5 times more likely than the general population to develop dependence on substances, including nicotine (Lee et al. 2011). Smoking and nicotine dependence are well-documented issues among individuals with ADHD, with research indicating that they are more likely to initiate smoking at an earlier age and become daily smokers compared to those without ADHD (Lee et al. 2011; Molina and Pelham 2003; Rhodes et al. 2016). Importantly, previous research has found that women with ADHD are at an increased risk of smoking both early and late in pregnancy, and more likely to continue smoking between these time points, suggesting challenges with cessation during critical periods in life, such as pregnancy (Andersson et al. 2020). Smoking during pregnancy is a serious and preventable risk factor associated with a range of negative outcomes for both the mother and the child. For instance, maternal smoking during pregnancy increases the likelihood of low birth weight, preterm birth, stillbirth, and is also linked to higher infant mortality (Gunnerbeck et al. 2023; Brinchmann et al. 2023). However, despite this well-established evidence on smoking, much less is known about the use of other nicotine products, such as snuff, among women with ADHD. Given the elevated risk of nicotine dependence in women with ADHD, it is important to investigate whether these individuals are more likely to use other forms of nicotine, such as snuff, during pregnancy.

Snuff, a smokeless form of tobacco that contains high levels of nicotine, is commonly used as an alternative to smoking, particularly in countries such as Sweden. Like maternal smoking during pregnancy, maternal snuff use during pregnancy is also linked to negative health outcomes, including premature birth and reduced birth weight (Brinchmann et al. 2023). Therefore, understanding the extent of snuff use among women diagnosed with ADHD is important. Moreover, the high prevalence of psychiatric comorbidities in individuals diagnosed with ADHD may further complicate the association between ADHD and nicotine use. Specifically, depression, anxiety, and SUDs are conditions that frequently co-occur with ADHD and are also known to be associated with a higher risk of nicotine use (Fluharty et al. 2016; Weinberger et al. 2016). These comorbidities may amplify the likelihood of nicotine dependence and pose additional challenges in cessation efforts. Thus, it is important to examine how the association between ADHD and snuff use may vary depending on the presence or absence of these common psychiatric disorders.

Although the relationship between ADHD and nicotine dependence has been well documented, little is known about the specific risk of snuff use among pregnant women diagnosed with ADHD. Given the growing public health concern regarding smokeless tobacco use and its impact during pregnancy, further investigation into snuff use among this high-risk group is warranted. Using Swedish population-based register data, this study aims to examine the association between ADHD and snuff use prior to and early in pregnancy, as well as persistent use between these time points. We also explore the associations between ADHD and snuff use in the presence of common psychiatric disorders: depression, anxiety, and/or SUDs. This will provide a more detailed understanding of how ADHD and psychiatric disorders influence snuff use patterns before and during early pregnancy.

The following research questions will be addressed:

1. To what extent are women diagnosed with ADHD more likely to use snuff prior to pregnancy, compared to women without ADHD?
2. To what extent are women diagnosed with ADHD more likely to use snuff early in pregnancy, compared to women without ADHD?
3. To what extent are women diagnosed with ADHD more likely to persist in snuff use from prior to pregnancy to early in pregnancy, compared to women without ADHD?
4. How does the association between ADHD and snuff use differ based on the presence of depression, anxiety and/or SUDs?

## Methods

### Study population

This study conforms to the STROBE (Strengthening the Reporting of Observational Studies in Epidemiology) reporting guideline. The Medical Birth Register in Sweden (MBR; Cnattingius et al. 2023) has systematically been collecting health data on all pregnancies in Sweden since its establishment in 1973. The register includes information on all live births in Sweden, irrespective of gestational age, as well as stillbirths from 22 completed gestational weeks onwards. Pregnancies not resulting in a live birth or stillbirth from 22 weeks (e.g., miscarriages and induced abortions before 22 weeks) are not included in the register (Cnattingius et al. 2023). In Sweden, each resident is assigned a unique personal identification number at birth or immigration (Ludvigsson et al. 2009), allowing for linkage across national registers and databases. We used the MBR to identify all singleton live births occurring between 2000 and 2020, yielding a total of 2,165,790 pregnancies from 1,231,578 women. Each pregnancy was treated as a separate observation, and information on snuff use was obtained at the first antenatal visit, reflecting use prior to and early in pregnancy rather than prospective follow-up throughout pregnancy.

This study was approved by the Swedish Ethical Review Authority (reference number 2020–06540).

## Measures

### ADHD

The Swedish National Patient Register (NPR; Ludvigsson et al. 2011) has recorded comprehensive data on all psychiatric inpatient care since 1987 and outpatient care since 2001. Diagnoses in this register are based on the Swedish versions of the International Classification of Diseases (ICD) including ICD 9 (1987 to 1996) and ICD-10 (since 1997). Additionally, the Swedish Prescribed Drug Register (PDR), established in 2005, contains detailed records of dispensed medications (Wettermark et al. 2007). To define ADHD status, we classified women as having ADHD if they had ever received an ADHD diagnosis in the NPR (International Classification of Diseases: ICD-9 code 314 or ICD-10 code F90) or had been prescribed ADHD-specific medication recorded in the PDR. The included medications, identified by Anatomical Therapeutic Chemical (ATC) codes, were methylphenidate (N06BA04), amphetamine (N06BA01), dexamphetamine (N06BA02), atomoxetine (N06BA09), lisdexamphetamine (N06BA12), and Guanfacine (C02AC02). Using this broad definition of life-time ADHD diagnosis, a total of 60,089 women (2.8%) were classified as having ADHD.

### Snuff

The MBR has recorded data on maternal snuff use at the first antenatal visit since 1999, typically during the first trimester, and at weeks 30–32 of pregnancy. During the first antenatal visit, midwives collect snuff use data through self-reported questionnaires, where women respond to questions pertaining to snuff use prior to pregnancy as well as snuff use at the time (i.e., early in pregnancy). These data are recorded in standardized medical records using predefined response options coded as binary variables (yes/no), and subsequently transferred to the Medical Birth Register (MBR). Due to a high proportion of missing data in recent years, snuff use in late pregnancy (week 30–32) is not reliable for research purposes (Cnattingius et al. 2023). Reports from the National Board of Health and Welfare indicate that since 2015, 70–80% of data for this variable is missing, with over 90% missing in most Swedish regions by 2019, due to a system error in the digital records. Therefore, we retrieved information on snuff use (yes/no) from the first antenatal visit, including snuff use prior to pregnancy as well as snuff use early in pregnancy. Information on snuff use at the first antenatal visit was available for approximately 95–96% of pregnancies, and with similar levels of missingness in women with and without ADHD. We also combined snuff use prior to pregnancy with snuff use early in pregnancy to retrieve information on persistence in snuff use. Women who used snuff both prior to pregnancy and early in pregnancy were defined as persistent snuff users. Persistent snuff use represents a subset of early pregnancy use, as it includes only women who reported snuff use both prior to and during early pregnancy.

### Psychiatric disorders

A comorbid psychiatric disorder was defined as a diagnosis of depression, anxiety, and/or SUDs at least one year before childbirth, identified using the NPR. For details on the included ICD codes, see Table S1 in Supplementary Material.

### Covariates

To account for potential period effects, we covaried for *year of childbirth*, retrieved from the MBR (2000–2020), modeled as a continuous variable. This adjustment is important, as both snuff use prevalence and diagnostic practices may have changed over time. As smokeless tobacco, such as snuff, is particularly popular among young individuals (Lipari and Van Horn 2017) we included *maternal age at childbirth* as a covariate, retrieved from the MBR, modeled as a continuous variable. Socioeconomic status (SES) is a relevant factor in both ADHD (Larsson et al. 2014) and nicotine use (Pennanen et al. 2014), with lower SES linked to higher rates of nicotine dependence. Thus, we covaried for SES, *using the highest achieved maternal education at the time of each childbirth as a proxy.* Educational data were retrieved from the Longitudinal Integration Database for Health Insurance and Labour Market Studies (LISA; Ludvigsson et al. 2019) in Sweden, categorizing maternal education into seven levels: less than 9 years, 9 years, 10–11 years, 12 years, 13–14 years, 15 years, and more than 15 years.

### Statistical analyses

Data management and descriptive analyses were conducted using SAS software version 9.4 (SAS Institute Inc., Cary, NC) and R (version 4.0.5). Descriptive characteristics of the study population were summarized using means and standard deviations (SD) for continuous variables and frequencies and percentages for categorical variables. Given that some women contributed with multiple observations over time (i.e., women giving birth multiple times between 2000 and 2020), we accounted for within-individual correlations by using robust standard errors clustered by ID.

As a first step, we assessed the prevalence of our three outcome variables: snuff use prior to and early in pregnancy, and persistent snuff use among women diagnosed with and without ADHD. Annual prevalence rates were calculated as the proportion of women reporting snuff use in each calendar year, stratified by ADHD status. In these analyses, ADHD status was defined using a lifetime approach and applied consistently across all calendar years, regardless of the timing of diagnosis in relation to pregnancy. The numerator consisted of the number of women reporting snuff use, and the denominator consisted of all women with available data on snuff use in that year. As a second step, we estimated odds ratios (ORs) and 95% confidence intervals (CIs) between ADHD and snuff use by fitting a series of logistic regressions using the stats package from base R. In these models, ADHD served as the predictor, while snuff use prior to and early in pregnancy as well as persistent use were the outcomes of interest.

Two models were fitted for each outcome: an unadjusted model and a model adjusted for year of childbirth, maternal age at childbirth, and maternal education. Observations with missing data on covariates included in the regression models were excluded from the analyses (complete-case analysis). Given the high comorbidity between ADHD and other psychiatric disorders (Chen et al. 2018; Kessler et al. 2006), we examined whether the association between ADHD and snuff use varied by the presence or absence of common psychiatric disorders, specifically depression, anxiety and/or SUDs. We used Wald test to statistically test differences in the associations.

#### Sensitivity analyses

*First*, we applied a more stringent definition of ADHD, requiring either an ADHD diagnosis or medication prescription to be recorded in the registers within the year prior to childbirth. Under this criterion, 20,493 (1.0%) women were classified as having ADHD before childbirth. This stricter definition was employed to ensure the correct temporal relationship between ADHD and the outcome of interest (snuff use), thereby minimizing the risk of misclassification bias and enhancing the robustness of our findings.

*Second*, women were defined as having ADHD if they had been diagnosed through the NPR, *n* = 53,822 (2.5%), thus excluding information from medication prescriptions. This definition was employed to assess whether the results would differ when considering only clinically diagnosed cases of ADHD. This approach helps minimize potential bias associated with including cases where ADHD might have been identified solely through medication use, which could have been for different indications. In this sensitivity analysis, ADHD diagnoses could be recorded at any time.

*Third*, we further refined the definition of ADHD by defining ADHD as having at least two diagnoses of ADHD recorded in the NPR, 51,345 (2.4%). This stricter criterion was applied to ensure the reliability of the ADHD diagnosis, as it requires more than one diagnostic entry, thus reducing the risk of misclassification and ensuring that the individuals classified as having ADHD had a more confirmed diagnosis. In this sensitivity analysis, no specific time frame between diagnoses was required, and ADHD diagnoses could be recorded at any time during the observation period, including before or after pregnancy.

*Fourth*, we restricted the cohort to include women who gave birth to their first child within the study period, resulting in 952,206 pregnancies. Of these, 28,120 (2.3%) women had a lifetime diagnosis of ADHD. This restriction was applied to examine the association between ADHD and snuff use among first-time mothers. By focusing on the first pregnancy, we aimed to reduce potential influence by previous pregnancy experiences, which could influence both ADHD diagnosis and snuff use behaviors.

Fifth, we conducted additional analyses stratified by calendar period (2000–2005, 2006–2010, 2011–2015, and 2016–2020) to examine whether the association between ADHD and snuff use varied over time. These analyses were performed to explore potential temporal trends in the strength of the associations and to assess whether the observed relationships differed across calendar periods.

## Results

Maternal characteristics of the study cohort are presented in Table 1. The mean age at giving birth within the study period was 27.6 years for women with ADHD and 30.4 years for women without ADHD. Clinically diagnosed depression was more prevalent among women with ADHD (24.6%) than those without ADHD (3.4%). Similarly, anxiety disorders and SUDs were more common in women diagnosed with ADHD, with prevalence rates of 25.7% and 18.6%, respectively, compared to 3.5% and 2.2% in women without ADHD.

**Table 1:** Maternal characteristics in women diagnosed with ADHD and without ADHD

Maternal characteristics	ADHD: Yes *n* = 60,089 (2.8) *n* (%)	ADHD: No *n* = 2,105,701 (97.2) *n* (%)
Mean age at childbirth	27.6 years (SD = 5.7)	30.4 years (SD = 5.1)
Distribution of births		
2000–2005	13,972 (23.2)	534,619 (25.4)
2006–2010	14,101 (23.5)	505,656 (24.0)
2011–2015	15,307 (25.5)	526,388 (25.0)
2016–2020	16,709 (27.8)	539,038 (25.6)
Maternal Education at childbirth		
< 9 years	499 (0.8)	44,351 (2.5)
9 years	21,664 (23.7)	174,149 (5.4)
10–11 years	8,275 (18.3)	200,984 (10.9)
12 years	17,147 (30.6)	587,734 (25.7)
13–14 years	4,729 (11.3)	284,529 (15.2)
15 years	5,598 (15.0)	671,160 (38.9)
> 15 years	53 (0.3)	13,099 (1.4)
*Missing*	*2*,*124 (3.1)*	*129*,*695 (5.9)*
Psychiatric disorders^1^		
Depression	14,780 (24.6)	70,792 (3.4)
Anxiety	15,420 (25.7)	74,334 (3.5)
SUDs	11,156 (18.6)	46,082 (2.2)
Snuff before pregnancy		
*Missing*	*2*,*722 (4.5)*	*86*,*580 (4.1)*
Snuff early in pregnancy		
*Missing*	*2*,*790 (4.6)*	*88*,*429 (4.2)*
Persistent snuff use		
*Missing*	2,947 (4.9)	93,100 (4.4)

Maternal education refers to the highest achieved maternal education at first pregnancy. ^1^Depression, anxiety, and/or SUDs are diagnosed at least one year prior to childbirth. Women may have more than one psychiatric disorder

### To what extent are women diagnosed with ADHD more likely to use snuff prior to pregnancy, compared to women without ADHD?

A total of 7.0% of women diagnosed with ADHD used snuff 3 months prior to pregnancy, compared to 3.5% in those without ADHD, equivalent to an unadjusted odds ratio (OR) of 2.11, 95% confidence interval (CI: 2.04–2.18). After adjusting for year of childbirth, maternal age at childbirth, and maternal education, women with ADHD were still more likely to use snuff 3 months prior to pregnancy adjOR = 1.89, 95% CI (1.83–1.95), compared to women without ADHD, see Table 2.

**Table 2:** Association between ADHD and snuff use in women diagnosed with ADHD compared to women without ADHD

	Women with ADHD		Women without ADHD			
	*N*	Prevalence %	*N*	Prevalence %	OR (95% CI)	OR^1^ (95% CI)
Total Population (*N* = 2,165,790)	**60,089**	**2.8**	**2,105,701**	**97.2**	-	-
Snuff use prior to pregnancy	4,210	7.0	73,138	3.5	2.11 (2.04–2.18)	1.89 (1.83–1.95)
Snuff use early in pregnancy	1,974	3.3	24,248	1.1	2.93 (2.80–3.07)	2.43 (2.32–2.55)
Persistent snuff use	1,631	2.7	20,191	1.0	2.90 (2.75–3.05)	2.39 (2.27–2.52)

^1^Adjusted for year of childbirth, maternal age at childbirth, and maternal education at first pregnancy

### To what extent are women diagnosed with ADHD more likely to use snuff early in pregnancy, compared to women without ADHD?

A total of 3.3% of the women with ADHD used snuff early in pregnancy, compared to 1.1% in those without ADHD, equivalent to an unadjusted OR of 2.93 95% CI (2.80–3.07). After adjusting for year of childbirth, maternal age at childbirth, and maternal education, women with ADHD were still more likely to use snuff early in pregnancy adjOR = 2.43, 95% CI (2.32–2.55), compared to women without ADHD, see Table 2.

### To what extent are women diagnosed with ADHD more likely to persist in snuff use from prior to pregnancy to early in pregnancy, compared to women without ADHD?

A total of 2.7% of women with ADHD persisted in snuff use between the two time points (3 months prior to pregnancy, and early in pregnancy), compared to 1.0% in women without ADHD, equivalent to an unadjusted OR of 2.90, 95% CI (2.75–3.05). After adjusting for year of childbirth, maternal age at childbirth, and maternal education, women with ADHD remained more likely to persist in snuff use adjOR = 2.39, 95% CI (2.27–2.52), compared to women without ADHD, see Table 2.

**Fig. 1:**
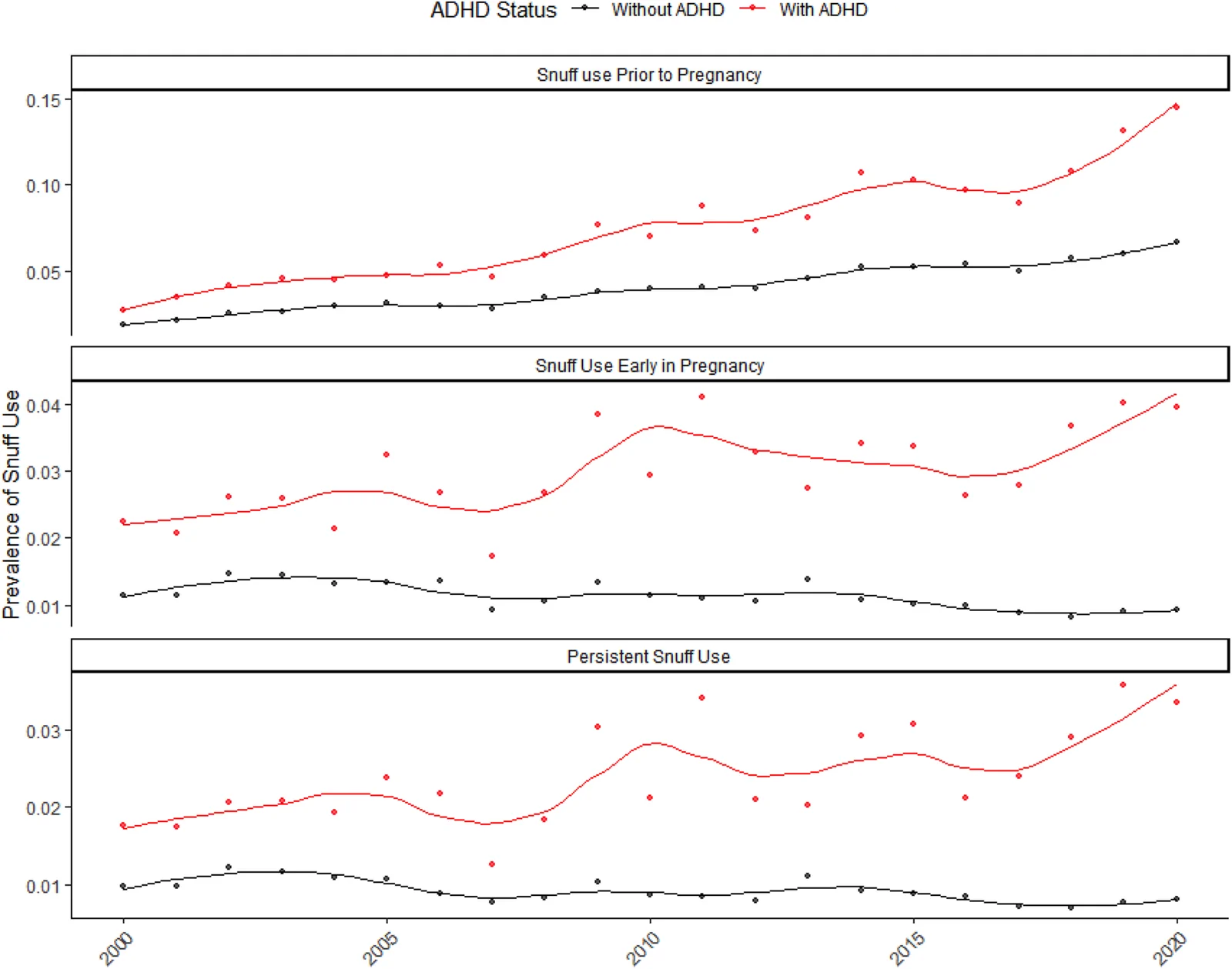
Prevalence of snuff use among women with and without ADHD

The prevalence of snuff use (prior to pregnancy, early in pregnancy, and persistent use) in women with and without ADHD over time is displayed in Fig. 1.

### How does the association between ADHD and snuff use differ based on the presence of depression, anxiety, and/or SUDs?

Among women *without* depression, anxiety, and/or SUDs, those diagnosed with ADHD had significantly higher odds of snuff use prior to pregnancy (adjOR = 1.76, 95% CI: 1.67–1.84), early in pregnancy (adjOR = 2.08, 95% CI: 1.94–2.23), and persistent snuff use (adjOR = 2.08, 95% CI: 1.94–2.23), compared to women without ADHD.

Among women *with* depression, anxiety, and/or SUDs, the associations between ADHD and snuff use remained statistically significant but were attenuated: prior to pregnancy (adjOR = 1.25, 95% CI: 1.19–1.31), early in pregnancy (adjOR = 1.48, 95% CI: 1.38–1.59), and persistent snuff use (adjOR = 1.42, 95% CI: 1.32–1.54), compared to women without ADHD. Wald tests indicated significantly weaker associations between ADHD and snuff use among women with depression, anxiety, and/or SUDs, compared to those without depression, anxiety, and/or SUDs, for snuff use prior to pregnancy, early in pregnancy, and persistent snuff use (all *p* < .001), see Table 3. For prevalence of snuff use by ADHD and comorbidity status, see Table S2 in supplementary material.

**Table 3:** Association between ADHD and snuff use in women diagnosed with ADHD compared to women without ADHD, stratified on the presence or absence of depression, anxiety, and/or SUDs

Women without depression, anxiety or SUDs (*n* = 1,992,086)	OR (95% CI)	OR^1^ (95% CI)	Women with depression, anxiety, or SUDs (*n* = 173,704)	OR (95% CI)	OR^1^ (95% CI)	*p*-value^2^
Snuff use prior to pregnancy	1.78 (1.69–1.86)	1.76 (1.67–1.84)	Snuff use prior to pregnancy	1.30 (1.24–1.36)	1.25 (1.19–1.31)	< 0.001
Snuff use early in pregnancy	2.45 (2.29–2.62)	2.08 (1.94–2.23)	Snuff use early in pregnancy	1.74 (1.63–1.87)	1.48 (1.38–1.59)	< 0.001
Persistent snuff use	2.46 (2.27–2.65)	2.08 (1.93–2.25)	Persistent snuff use	1.68 (1.55–1.80)	1.42 (1.32–1.54)	< 0.001

Total *N* = 2,165,790. ^1^Adjusted for year of childbirth, maternal age at childbirth, and maternal education at first pregnancy. ^2^p-value from Wald test, comparing the adjusted OR estimates for women with ADHD, stratified by the presence or absence of depression, anxiety, and/or SUDs

### Sensitivity analyses

To assess the robustness of our estimates and eliminate potential alternative explanations for the observed associations, we performed a series of sensitivity analyses. First, using a stricter definition of ADHD, requiring that an ADHD diagnosis or prescription for ADHD medication to be recorded in the registers within the year preceding childbirth, the results remained consistent. In fully adjusted models, compared to women without ADHD, women with ADHD were more likely to use snuff both prior to pregnancy (adjOR = 1.96, 95% CI: 1.87–2.06) and early in pregnancy (adjOR = 2.75, 95% CI: 2.55–2.95). Additionally, women with ADHD were more likely to continue using snuff from prior to pregnancy to early in pregnancy (adjOR = 2.69, 95% CI: 2.48–2.91), compared to women without ADHD. Crude and stepwise adjusted models can be found in supplementary Table S3.

Second, in the sensitivity analysis where women were defined as having ADHD **only** if they had been diagnosed through the NPR, revealed similar results. In fully adjusted models, compared to women without ADHD, women with ADHD were more likely to use snuff both prior to pregnancy (adjOR = 1.92, 95% CI: 1.85–1.98) and early in pregnancy (adjOR = 2.48, 95% CI: 2.36–2.60). Additionally, women with ADHD were more likely to continue using snuff from prior to pregnancy to early in pregnancy (adjOR = 2.44, 95% CI: 2.31–2.57), compared to women without ADHD. Crude and stepwise adjusted models can be found in supplementary Table S4.

Third, in the sensitivity analysis where we only included women with at least two diagnoses of ADHD recorded in the NPR, results remained largely consistent with previous findings. In fully adjusted models, compared to women without ADHD, women with ADHD were more likely to use snuff both prior to pregnancy (adjOR = 1.92, 95% CI: 1.85–1.98) and early in pregnancy (adjOR = 2.48, 95% CI: 2.35–2.60). Additionally, women with ADHD were more likely to continue using snuff from prior to pregnancy to early in pregnancy (adjOR = 2.43, 95% CI: 2.30–2.57), compared to women without ADHD. Crude and stepwise adjusted models can be found in supplementary Table S5.

Fourth, in the sensitivity analysis where we only included women giving birth to their first child within the study period, results remained consistent with previous findings. In fully adjusted models, compared to women without ADHD, women with ADHD were more likely to use snuff both prior to pregnancy (adjOR = 1.68, 95% CI: 1.61–1.76), and early in pregnancy (adjOR = 2.27, 95% CI: 2.10–2.46). Additionally, women with ADHD were more likely to continue using snuff from prior to pregnancy to early in pregnancy (adjOR = 2.18, 95% CI: 2.00-2.36), compared to women without ADHD. Crude and stepwise adjusted models can be found in supplementary Table S6.

Fifth, in the sensitivity analysis stratified by calendar period, the association between ADHD and snuff use showed an increasing trend over time. This pattern was observed across all outcomes but was most pronounced for snuff use early in pregnancy and persistent snuff use, where the adjusted odds ratios increased across successive calendar periods. Detailed estimates are presented in supplementary Table S7.

## Discussion

To our knowledge, this is the first large-scale population-based study to investigate the association between ADHD and snuff use prior to and early in pregnancy. By addressing this gap, our findings may have important implications for public health interventions targeting nicotine use in women with ADHD. Given the potential health risks associated with snuff use during pregnancy, these insights could help guide tailored prevention strategies for this vulnerable population. Consistent with prior research demonstrating an increased risk of nicotine dependence among individuals with ADHD (Lee et al. 2011; Rhodes et al. 2016) and an increased risk of smoking during pregnancy among women diagnosed with ADHD (Andersson et al. 2020), we found that women diagnosed with ADHD were significantly more likely to use snuff prior to and early in pregnancy, as well as persist in snuff use between these time periods. Stratified analyses showed that the associations between ADHD and snuff use were strongest among women diagnosed with ADHD in the absence of depression, anxiety, and/or SUDs. These findings suggest that psychiatric comorbidities may influence the strength of the association between ADHD and snuff use prior to and early in pregnancy.

### ADHD and snuff use prior to pregnancy

Women diagnosed with ADHD were twice as likely to use snuff prior to pregnancy compared to women without ADHD. This finding aligns with research on the link between ADHD and nicotine use, which has suggested that impulsiveness, reward system dysregulation, and self-medication motives may contribute to increased substance use (Mansvelder and McGehee 2002). The self-medication hypothesis suggests that individuals with ADHD may use nicotine to alleviate symptoms of inattention, impulsivity, and hyperactivity (Castle et al. 2007; Levin et al. 2001). Given that nicotine modulates dopamine function, often dysregulated in ADHD, its use may temporarily improve attention and emotional regulation. This could partially explain why women with ADHD are at an increased risk of snuff use prior to pregnancy, as they may use it to manage cognitive and emotional dysregulation.

### ADHD and snuff use early in pregnancy

The increased likelihood of snuff use early in pregnancy among women diagnosed with ADHD is concerning, given the established adverse health effects of nicotine exposure during gestation (Brinchmann et al. 2023; Gunnerbeck et al. 2023). While previous studies have documented higher rates of cigarette smoking in pregnant women with ADHD (Andersson et al. 2020), our findings extend this knowledge by demonstrating a similar pattern for snuff use. The indication of a stronger association (non-overlapping CIs) between ADHD and early pregnancy snuff use, compared to pre-pregnancy use, suggests that factors beyond general nicotine dependence may contribute to this behavior. Possible explanations include heightened stress sensitivity and difficulties with emotion regulation, which may be exacerbated by the hormonal changes of pregnancy. The self-medication hypothesis further suggests that women with ADHD may turn to nicotine use to manage these challenges, as nicotine has been shown to temporarily alleviate ADHD-related cognitive difficulties and emotional dysregulation. This could contribute to continued snuff use despite the known risks during pregnancy.

### Persistence in snuff use from pre-pregnancy to early pregnancy

It is important to note that persistent snuff use represents a subset of early pregnancy use, as all persistent users reported snuff use both prior to and during early pregnancy, whereas early pregnancy use also includes women who initiated use during pregnancy. While early pregnancy use captures any use during pregnancy, persistent use reflects continued use across the transition into pregnancy and may indicate greater difficulty with cessation. Descriptive analyses further support this interpretation, as a higher proportion of women with ADHD who used snuff prior to pregnancy continued use into early pregnancy compared to women without ADHD (approximately 39% vs. 28%). Persistence in nicotine use during pregnancy is particularly concerning due to the cumulative exposure risks for the fetus. Our findings indicate that women diagnosed with ADHD were significantly more likely to persist in snuff use from prior to pregnancy to early in pregnancy compared to their non-ADHD counterparts. This is in line with previous research suggesting that differences in smoking cessation may be the result of greater withdrawal severity in individuals with ADHD compared to those without ADHD (Pomerleau et al. 1995). This suggests that ADHD is an independent risk factor for continued nicotine use. This pattern mirrors prior research on smoking behaviors in pregnant women with ADHD, where lower cessation rates have been reported (Andersson et al. 2020). The mechanisms underlying this persistence may include difficulties with cessation, driven by impulsiveness and challenges with self-regulation, both hallmarks of ADHD (Levin et al. 2001). The reinforcing effects of nicotine on the dopamine system may further complicate cessation efforts, as the immediate mood-lifting effects of nicotine can create a cycle of dependence, making it harder for women diagnosed with ADHD to quit. Prior research has shown that lifetime prevalence of mood and anxiety disorders is significantly higher among women than men (Conway et al. 2006), which may contribute to a heightened susceptibility to tobacco use, misuse, and dependence in this population.

### Stratification by depression, anxiety, and/or SUDs

The association between ADHD and snuff use was weaker among women with depression, anxiety, and/or SUDS, compared to those without depression, anxiety, and/or SUDs. It is important to interpret this attenuation in the context of a higher baseline prevalence of snuff use in women with comorbid psychiatric disorders, including among those without ADHD. As shown in Table S2, snuff use is more common in women with depression, anxiety, and/or SUDs regardless of ADHD status, which reduces the relative difference between groups and consequently attenuates the observed odds ratios. However, the absolute prevalence of snuff use remains higher in this group, highlighting a substantial public health burden. One possible explanation is that women entering pregnancy with multiple psychiatric disorders may receive more structured support from healthcare services. This enhanced support could facilitate better access to the treatment of psychiatric disorders which could potentially lower the use of snuff among this group. In Sweden, some regions have made efforts to improve psychological support for vulnerable groups, including pregnant women with mental health issues, by implementing initiatives such as supportive counseling, structured identification of depression and anxiety, and more accessible healthcare pathways (Socialstyrelsen, 2024). These initiatives aim to provide coordinated, multiprofessional care, which is crucial for improving mental health outcomes and supporting more effective coping during pregnancy. However, further longitudinal research is needed to clarify the mechanisms underlying these associations and to understand how ADHD and co-occurring psychiatric disorders interact over time, particularly in the context of pregnancy.

### Clinical and public health implications

Given that snuff use during pregnancy is associated with adverse birth outcomes, including increased risks of stillbirth and neonatal complications (Brinchmann et al. 2023; England et al. 2003), our findings highlight the need for targeted public health interventions. Women with ADHD may require tailored smoking and snuff cessation programs that account for their specific cognitive and behavioral challenges. Additionally, healthcare providers should be aware of the heightened risk of snuff use in pregnant women diagnosed with ADHD and proactively address nicotine cessation in prenatal care settings. Importantly, the increasing trend in the association between ADHD and snuff use over calendar time suggests that disparities between women with and without ADHD may be widening, particularly for continued use during pregnancy. This highlights the need for timely and targeted interventions to prevent further nicotine-related health inequalities in this population.

### Strengths and limitations

A key strength of this study is the use of a large, population-based cohort with high-quality registry data, allowing for robust statistical analyses. Additionally, the use of multiple sensitivity analyses strengthens the validity of our findings. However, some limitations should be acknowledged.

First, although snuff use was self-reported, which may introduce recall or social desirability bias, previous research has shown a high agreement between smoking information in MBR and maternal serum cotinine levels (Mattsson et al. 2016). It is likely that this may also apply to snuff use; however, future research could further explore this to ensure that snuff use is reliably recorded in Swedish population-based registers. In addition, due to a high proportion of missing data, we were unable to examine snuff use in late pregnancy (week 30–32), a crucial period for fetal development. Reports from the National Board of Health and Welfare indicate that since 2015, 70–80% of data for this variable have been missing, with over 90% missing in many Swedish regions by 2019, primarily due to a system error in the digital records. This limitation prevents us from fully understanding the association between ADHD and snuff use throughout pregnancy.

Second, we did not examine overlap with other nicotine products, such as smoking. While the association between ADHD and smoking during pregnancy has been well established, including in Swedish register-based studies, patterns of use may differ between nicotine products. Snuff use represents a distinct form of nicotine exposure, with different social norms, perceived risks, and patterns of use during pregnancy. Future research should investigate patterns of dual use and switching between nicotine products among women with ADHD.

Third, there may be residual confounding due to unmeasured factors, such as the severity of ADHD or adherence to ADHD treatment. These factors could still influence the likelihood of snuff use and, as they were not accounted for in our models, may lead to some degree of bias in the results. For example, women with more severe ADHD symptoms may be more prone to using substances like snuff as a form of self-medication, and those with lower adherence to treatment may be less able to manage impulsive behaviors. Also, it is important to mention that the use of clinical diagnoses of ADHD in the Swedish registers likely captures the more severe ADHD cases. In addition, in the sensitivity analysis requiring at least two ADHD diagnoses, no restrictions were placed on the timing between diagnoses. As a result, some women may have been classified as having ADHD based on diagnoses recorded after pregnancy, which may introduce temporal ambiguity. However, this approach was intended to increase diagnostic specificity, and the consistency of results across sensitivity analyses suggests that this is unlikely to have materially influenced the findings.

Fourth, our stratified models included the tree most common psychiatric disorders in women diagnosed with ADHD (i.e., depression, anxiety, and SUDs) (Fluharty et al. 2016; Weinberger et al. 2016). However, we acknowledge that other psychiatric disorders, such as personality disorders, may also be present in the cohort and potentially influence the observed estimates. Also, although comorbid psychiatric disorders were defined prior to childbirth in a way that ensured temporal precedence relative to snuff use in early pregnancy, there was less temporal separation in relation to snuff use prior to pregnancy, which may introduce some temporal ambiguity for that outcome.

Fifth, our study only includes data up until 2020, and recent years have seen an increase in snuff use, both in the general population and, particularly among women with ADHD (Public Health Agency of Sweden, 2024a, 2024b). This trend is also reflected in our descriptive results (i.e., Fig. 1). As a result, the findings from this study may not fully generalize to more recent cohorts, given the rise in snuff use since the data was collected. Therefore, the true association between ADHD and snuff use during pregnancy could be underestimated. In addition, Fig. 1 suggests that the gap in smoking prevalence between women diagnosed with and without ADHD is widening over time, with women with ADHD showing a stronger increase in smoking rates. This may reflect a growing burden of nicotine dependence within this population over time.

Sixth, we found that women with ADHD were more likely to continue using snuff between the two time points (prior to and early in pregnancy) compared to women without ADHD. We assumed that women who reported snuff use at both time points continued using it in between. Although we lacked information on any periods without use, we believe that more detailed data would not have altered the conclusion that women with ADHD are more likely to use snuff both before and early in pregnancy.

Seventh, our results are most relevant to countries where snuff is legally sold and widely used. However, the generalizability of these findings to other countries and cultures with different regulatory environments and nicotine product use patterns remains uncertain. Future research should explore whether similar patterns are observed with other nicotine products (e.g., e-cigarettes) in different cultural and regulatory contexts.

## Conclusions

This study is the first study to our knowledge to show that women diagnosed with ADHD are at an increased risk of snuff use prior to and early in pregnancy. These findings underscore the need for enhanced assessment and intervention efforts targeting nicotine use in this vulnerable population as part of comprehensive integrated care. Future research should explore underlying mechanisms driving this association and evaluate the possible effectiveness of ADHD-specific snuff-cessation strategies in pregnant women. By addressing these factors, we can begin to develop more effective interventions for preventing snuff use and mitigating its impact on both maternal and fetal health.

## Supplementary Information

Below is the link to the electronic supplementary material.

Supplementary Material 1

## Data Availability

Data cannot be shared publicly because of the Swedish Secrecy Act. Data from the Medical Birth Register, the Multi-Generation Register and the National Patient Register were used for this study and made available by ethical approval. Researchers may apply for access through the Swedish Research Ethics Boards (www.etikprovningsmyndigheten.se) and from the primary data owners Statistics Sweden (www.scb.se) and the National Board of Health and Welfare (www.socialstyrelsen.se), in accordance with Swedish law.

## Abbreviations

ADHD: Attention-Deficit/Hyperactivity Disorder
OR: Odds Ratio
SUD: Substance Use Disorder
