# Supplementary material for: Attention-deficit/hyperactivity disorder and snuff use prior to and early in pregnancy
Source: Arch Womens Ment Health. 2026 Jul 1;29(4):101. doi: 10.1007/s00737-026-01742-x (PMC13323211; doi:10.1007/s00737-026-01742-x)
Supplement: Supplementary file 1 — Supplementary Material 1 [file 737_2026_1742_MOESM1_ESM.docx]

Table S1. ICD-9 and ICD-10 Codes for Depression, Anxiety, and Substance Use Disorders (SUDs).

| **Disorder Category** | **ICD-9 Codes** | **ICD-10 Codes** |
| --- | --- | --- |
| **Depression** | 296B, 298A, 296W, 311X, 300E, 300F, 309A, 309B | F32, F33, F34, F381, F488, F4321, F530 |
| **Anxiety** | 300A, 300C, 300D, 313A | F40, F41 |
| **Substance Use Disorders (SUDs)** | 291, 303, 304, 305, 303X | F10, F11, F12, F13, F14, F15, F16, F17, F18, F19 |

Note. Depression, anxiety, and/or SUDs were defined as present if diagnosed at least one year before childbirth.

Table S2. Prevalence of snuff use prior to pregnancy, early in pregnancy, and persistent snuff use: by ADHD and Comorbidity Status.

|  | **ADHD (N)** | **(%)** | **No ADHD (N)** | **(%)** |
| --- | --- | --- | --- | --- |
| **Women without depression, anxiety, and/or SUDs**  (n = 1,992,086) | 34,284 | 1.7 | 1,957,802 | 98.3 |
| Snuff use prior to pregnancy | 1,883 | 5.5 | 62,572 | 3.2 |
| Snuff use early in pregnancy | 858 | 2.5 | 20,490 | 1.0 |
| Persistent snuff use | 714 | 2.1 | 16,988 | 0.9 |
| **Women with depression, anxiety, and/or SUDs**  (n = 173,704) | 25,805 | 14.9 | 147,899 | 85.1 |
| Snuff use prior to pregnancy | 2,327 | 9.4 | 10,566 | 7.4 |
| Snuff use early in pregnancy | 1,116 | 4.5 | 3,758 | 2.6 |
| Persistent snuff use | 917 | 3.7 | 3,203 | 2.2 |

Note. Prevalence estimates are calculated for the total study period (2000–2020) and represent the proportion (%) of pregnancies with reported snuff use within each subgroup.

Table S3. Association between ADHD and snuff use in women diagnosed with ADHD compared to women without ADHD.
Stricter definition of ADHD (one year preceding childbirth).

|  | **Prevalence of snuff use** | | **Crude ORs** | **Adjusted^1^ ORs** |
| --- | --- | --- | --- | --- |
|  | **ADHD (20,493)** | **Non-ADHD (2,145,297)** | **OR (95% CIs)** | **OR (95% CIs)** |
| **Snuff use prior to pregnancy** | 1,969 (9.6) | 75,379 (3.5) | 2.91 (2.78–3.05) | 1.96 (1.87-2.06) |
| **Snuff use early in pregnancy** | 820 (4.0) | 25,402 (1.2) | 3.46 (3.22-3.72) | 2.75 (2.55-2.95) |
| **Persistent snuff use** | 685 (3.3) | 21,137 (1.0) | 3.46 (3.20-3.73) | 2.69 (2.48-2.91) |

Note. Adjusted^1^ refers to; adjusted for year of childbirth, maternal age at childbirth, and maternal education. Prevalence estimates are calculated for the total study period (2000–2020) and represent the proportion (%) of pregnancies with reported snuff use within each subgroup.

Table S4. Association between ADHD and snuff use in women diagnosed with ADHD compared to women without ADHD.
Only including ADHD diagnoses from the NPR.

|  | **Prevalence of snuff use** | | **Crude ORs** | **Adjusted^1^ ORs** |
| --- | --- | --- | --- | --- |
|  | **ADHD (53,822)** | **Non-ADHD (2,111,968)** | **OR (95% CIs)** | **OR (95% CIs)** |
| **Snuff use prior to pregnancy** | 3,874 (7.2) | 73,474 (3.5) | 2.17 (2.09-2.24) | 1.92 (1.85-1.98) |
| **Snuff use early in pregnancy** | 1,827 (3.4) | 24,395 (1.2) | 3.02 (2.88-3.17) | 2.48 (2.36-2.60) |
| **Persistent snuff use** | 1,512 (2.8) | 20,310 (1.0) | 2.99 (2.84-3.16) | 2.44 (2.31-2.57) |

Note. Adjusted^1^ refers to; adjusted for year of childbirth, maternal age at childbirth, and maternal education. Prevalence estimates are calculated for the total study period (2000–2020) and represent the proportion (%) of pregnancies with reported snuff use within each subgroup.

Table S5. Association between ADHD and snuff use in women diagnosed with ADHD compared to women without ADHD.
Only including women with at least two ADHD diagnoses from the NPR.

|  | **Prevalence of snuff use** | | **Crude ORs** | **Adjusted^1^ ORs** |
| --- | --- | --- | --- | --- |
|  | **ADHD (51,345)** | **Non-ADHD (2,114,445)** | **OR (95% CIs)** | **OR (95% CIs)** |
| **Snuff use prior to pregnancy** | 3,693 (7.2) | 73,655 (3.5) | 2.16 (2.09-2.24) | 1.92 (1.85-1.98) |
| **Snuff use early in pregnancy** | 1,745 (3.4) | 24,477 (1.2) % | 3.02 (2.88-3.17) | 2.48 (2.35-2.60) |
| **Persistent snuff use** | 1,439 (2.8) | 20,383 (1.0) % | 2.98 (2.82-3.15) | 2.43 (2.30-2.57) |

Note. Adjusted^1^ refers to; adjusted for year of childbirth, maternal age at childbirth, and maternal education. Prevalence estimates are calculated for the total study period (2000–2020) and represent the proportion (%) of pregnancies with reported snuff use within each subgroup.

Table S6. Association between ADHD and snuff use in women diagnosed with ADHD compared to women without ADHD.
Only including the first pregnancy (parity = 1).

|  | **Prevalence of snuff use** | | **Crude ORs** | **Adjusted^1^ ORs** |
| --- | --- | --- | --- | --- |
|  | **ADHD (28,120)** | **Non-ADHD (924,086)** | **OR (95% CIs)** | **OR (95% CIs)** |
| **Snuff use prior to pregnancy** | 2,159 (7.7) | 39,330 (4.3) | 1.88 (1.80-1.97) | 1.68 (1.61-1.76) |
| **Snuff use early in pregnancy** | 800 (2.8) | 9,877 (1.1) | 2.72 (2.53-2.93) | 2.27 (2.10-2.46) |
| **Persistent snuff use** | 647 (2.3) | 7,981 (0.9) | 2.71 (2.50-2.94) | 2.18 (2.00-2.36) |

Note. Adjusted^1^ refers to; adjusted for year of childbirth, maternal age at childbirth, and maternal education. Prevalence estimates are calculated for the total study period (2000–2020) and represent the proportion (%) of pregnancies with reported snuff use within each subgroup.

**Table S7. Association between ADHD and snuff use, stratified by calendar period.**

| **Outcome** | **Calendar period** | **OR 95% CI** | **OR^1^ (95% CI)** |
| --- | --- | --- | --- |
| **Snuff use prior to pregnancy** | 2000–2005 | 1.66 (1.50-1.83) | 1.66 (1.50-1.83) |
|  | 2006–2010 | 1.96 (1.81-2.12) | 1.72 (1.59-1.87) |
|  | 2011–2015 | 2.16 (2.02-2.30) | 1.90 (1.77-2.03) |
|  | 2016–2020 | 2.26 (2.14-2.38) | 2.07 (1.96-2.19) |
| **Snuff use early in pregnancy** | 2000–2005 | 2.01 (1.78-2.27) | 1.88 (1.66-2.13) |
|  | 2006–2010 | 2.53 (2.26-2.83) | 2.10 (1.88-2.36) |
|  | 2011–2015 | 3.13 (2.84-3.44) | 2.46 (2.23-2.71) |
|  | 2016–2020 | 3.97 (3.65-4.32) | 3.10 (2.84-3.38) |
| **Persistent snuff use** | 2000–2005 | 1.93 (1.69-2.21) | 1.83 (1.60-2.10) |
|  | 2006–2010 | 2.53 (2.23-2.87) | 2.07 (1.82-2.35) |
|  | 2011–2015 | 3.08 (2.77-3.42) | 2.39 (2.15-2.67) |
|  | 2016–2020 | 3.89 (3.56-4.26) | 3.05 (2.77-3.35) |

Note. Adjusted^1^ refers to; adjusted for maternal age at childbirth and maternal education at first
pregnancy; models were stratified by calendar period.
